# Antagonistic Pleiotropy and Fitness Trade-Offs Reveal Specialist and Generalist Traits in Strains of Canine Distemper Virus

**DOI:** 10.1371/journal.pone.0050955

**Published:** 2012-12-11

**Authors:** Veljko M. Nikolin, Klaus Osterrieder, Veronika von Messling, Heribert Hofer, Danielle Anderson, Edward Dubovi, Edgar Brunner, Marion L. East

**Affiliations:** 1 Evolutionary Ecology, Leibniz Institute for Zoo and Wildlife Research-Berlin, Berlin, Germany; 2 INRS-Institut Armand-Frappier, Quebec, Canada; 3 Institut für Virologie, Freie Universität Berlin, Berlin, Germany; 4 Emerging Infectious Diseases Program, Duke-NUS Graduate Medical School, Singapore; 5 Animal Health Diagnostic Center, Cornell University, Ithaca, New York, United States of America; 6 Department of Medical Statistics, University of Göttingen, Göttingen, Germany; The University of Hong Kong, Hong Kong

## Abstract

Theoretically, homogeneous environments favor the evolution of specialists whereas heterogeneous environments favor generalists. Canine distemper is a multi-host carnivore disease caused by canine distemper virus (CDV). The described cell receptor of CDV is SLAM (CD150). Attachment of CDV hemagglutinin protein (CDV-H) to this receptor facilitates fusion and virus entry in cooperation with the fusion protein (CDV-F). We investigated whether CDV strains co-evolved in the large, homogeneous domestic dog population exhibited specialist traits, and strains adapted to the heterogeneous environment of smaller populations of different carnivores exhibited generalist traits. Comparison of amino acid sequences of the SLAM binding region revealed higher similarity between sequences from Canidae species than to sequences from other carnivore families. Using an *in vitro* assay, we quantified syncytia formation mediated by CDV-H proteins from dog and non-dog CDV strains in cells expressing dog, lion or cat SLAM. CDV-H proteins from dog strains produced significantly higher values with cells expressing dog SLAM than with cells expressing lion or cat SLAM. CDV-H proteins from strains of non-dog species produced similar values in all three cell types, but lower values in cells expressing dog SLAM than the values obtained for CDV-H proteins from dog strains. By experimentally changing one amino acid (Y549H) in the CDV-H protein of one dog strain we decreased expression of specialist traits and increased expression of generalist traits, thereby confirming its functional importance. A virus titer assay demonstrated that dog strains produced higher titers in cells expressing dog SLAM than cells expressing SLAM of non-dog hosts, which suggested possible fitness benefits of specialization post-cell entry. We provide *in vitro* evidence for the expression of specialist and generalist traits by CDV strains, and fitness trade-offs across carnivore host environments caused by antagonistic pleiotropy. These findings extend knowledge on CDV molecular epidemiology of particular relevance to wild carnivores.

## Introduction

Theoretical [1]–[3] and empirical evidence [4]–[9] suggests that organisms evolving in homogeneous environments tend to be more specialized than those evolving in heterogeneous environments. Specialists are expected to have reduced fitness in heterogeneous environments whereas the fitness of generalists in homogeneous environments is expected to be below that of a specialist. Such fitness trade-offs may be caused by antagonistic pleiotropy, in which a beneficial mutation in one environment is either harmful or neutral in another environment, or mutations that are neutral in the environment in which they arose are deleterious in another [3], [5], [8]. Specialists are thought to evolve more rapidly than generalists because selection for beneficial traits and against harmful traits is expected to occur more rapidly in homogeneous environments [1], [10].

Much of our knowledge of the evolution of specialists and generalists comes from studies of microorganisms, including viruses [7]–[9], [11]–[13]. Some viruses are described as specialists if they infect one or only a few closely related host species whereas others are viewed as generalists if they infect multiple hosts including species from different taxonomic groups [11]–[15]. On a finer scale, a generalist multi-host virus is likely to exist as several distinct genetic strains that circulate in overlapping but distinct ranges of host species [16]–[19]. Should particular strains of a multi-host virus evolve in one (of several possible) host species, they are expected to experience strong co-evolution and thus become specialist strains with increased fitness in the host to which they are adapted. Strains of the same multi-host virus that evolve in heterogeneous host environments composed of several species would experience weaker co-evolution and become generalist strains [5], [7], [8], [11], [13]. The fitness of generalist strains should be higher than that of specialist strains in all host species except for the particular species that specialist strains were adapted to. Thus, specialist and generalist strains of a multi-host virus are expected to show trade-offs in their relative fitness in different host environments.

Anthropogenic activities have radically changed the density of host species with profound consequences for the epidemiology of pathogens [20]. High densities of domesticated species have created extensive homogeneous host environments that favor strong host-specific selection and the evolution of specialist virus strains. Habitats less impacted by human activities containing lower density populations of several potential host species provide a heterogeneous host environment favorable to the evolution of generalist strains.

For many viruses, host range is determined by a variety of mechanisms, including the molecular mechanisms by which the virus gains entry to the cells of its host species [21]–[26]. The first step in this process involves the binding of virus surface proteins to host cell receptors. Strong co-evolution should produce receptor-binding proteins in specialist virus strains that are best adapted to the receptors of the matching host species, whereas weak co-evolution should favor surface proteins that bind equally well to the receptors of a wider range of host species.

Canine distemper virus (CDV) in the genus *Morbillivirus* and the family Paramyxoviridae is a multi-host pathogen of aquatic and terrestrial species in the order Carnivora [27]. Between 55 and 60 million years ago this order diverged into two suborders, the Feliformia and Caniformia [28]. Outbreaks of CDV have occurred in species within both the Caniformia, such as the domestic dog (*Canis familiaris*), African wild dog (*Lycaon pictus*) black-footed ferret (*Mustela nigripes*) and Baikal seal (*Pusa sibirica*), and Feliformia, such as the African lion (*Panthera leo*) and spotted hyena (*Crocuta crocuta*) [29]–[33]. Sensitivity to CDV varies greatly, even among species within one family. For example, in the Felidae, domestic cats (*Felis catus*) exposed to CDV seroconvert but do not develop clinical disease [34], [35], whereas large felids both seroconvert and develop clinical disease [29], [31].

CDV encodes two envelope glycoproteins, the hemagglutinin (CDV-H) protein which mediates virus binding to the target host cell receptor, and the fusion (CDV-F) protein which accomplishes membrane fusion, thereby enabling entry of the viral replication complex into the cytoplasm [36]. Interestingly, the CDV-H protein is the most variable H protein among all members of the genus *Morbillivirus*, which may explain why CDV has a far broader host range than other morbilliviruses [37]. In cultured cells, infection frequently leads to the formation of multinucleated cells known as syncytia. The proficiency of syncytium formation is chiefly determined by the CDV-H protein [38]. Currently, the only described [39] cellular receptor for the CDV-H protein on host lymphatic cells is the signaling lymphocytic activation molecule (SLAM, CD150). In humans it is expressed on immature thymocytes, memory T cells, and a subset of B cells and is rapidly induced in a wide range of immune cells after activation [40]–[42]. The importance of SLAM binding for CDV pathogenesis was experimentally demonstrated [43]. Measles virus (another *Morbillivirus*) also binds to SLAM, CD150 [40]–[42] and some strains are known to bind to cell receptor CD46 in both epithelial and immune cells [44]. Although it is unlikely that the CDV-H protein binds to CD46 [43], other as yet unidentified receptors are thought to exist on lymphatic and epithelial cells [45].

It is unclear to what extent the molecular structure of SLAM receptors of species belonging to different carnivore families alters the binding ability of CDV-H proteins of different strains. Recently it was proposed that the specificity of the CDV-H protein-SLAM receptor interaction represents a potential determinant of the host range [46]–[48]. In particular, it was hypothesized that strong positive selection at site 549 in the SLAM binding region of the CDV-H protein associated with the substitution of tyrosine (Y) by histidine (H) at this site changed CDV-H protein structure leading to the spread of CDV from domestic dogs to non-dog species [46]. Current information on the amino acid at site 549 in CDV strains worldwide suggests that positive selection at site 549 may be driven by adaptation of strains to their host species, given that the vast majority of domestic dog strains (and strains from other species in the Canidae family) have Y at this site, whereas strains from species in other carnivore families mostly have H at this site [49].

The CDV-H protein-host SLAM binding mechanism provides a useful cellular system to test whether stronger co-evolution between CDV and its most abundant host has produced strains that exhibit specialist traits in the globally large domestic dog population that persists at high densities in many human altered habitats [50], and generalist traits in CDV strains in other carnivore species in habitats containing several potential wildlife hosts and few or no domestic dogs [30], [31], [51]. This idea is plausible, given the presence of genetically distinct wildlife lineages of CDV, most notably in non-canid species in Europe [49], [52], [53] and genetic differences between CDV strains in domestic dogs and wild carnivores in relation to the residue at site 549 in the CDV-H protein [46], [49], but to our knowledge has not been tested.

To test this idea we predicted that amino acid sequences of the entire SLAM binding region of species in the Canidae (including the domestic dog) should be more similar to each other than to sequences from species in other carnivore families, which our results confirmed. We then tested the predicted expression of specialist traits and generalist traits (detailed in Table 1) both at syncytia formation and virus production using two *in vitro* assays. The syncytia formation assay measured a parameter relevant to cell entry, i.e., the mean number of nuclei per syncytium formed by CDV-H proteins from domestic dog and non-dog CDV strains in cells expressing SLAM from the domestic dog, SLAM from the African lion and SLAM from the domestic cat. The virus titer assay measured virus production post cell entry by the same CDV strains in cells expressing SLAM from the same three carnivore species. Both assays provided results consistent with the predicted expression of specialist traits by CDV-H proteins and strains from domestic dog in cells expressing domestic dog SLAM and generalist traits by CDV-H proteins and strains from non-dog hosts regardless of which species SLAM was expressed on cells. Finally, we investigated antagonistic pleiotropic effects of the residue at site 549 in the CDV-H protein in different host receptor environments. We predicted that experimental substitution of residue Y, characteristic of domestic dog CDV strains at site 549, with residue H, typical for CDV strains from non-dog hosts [46], [49], in the CDV-H protein from one domestic dog strain would decrease expression of specialist traits in the syncytia formation assay, which is what our results showed.

**Table 1:** Predicted expression of specialist and generalist traits by canine distemper virus strains in relation to different host species receptors (SLAM CD150).

CDV strain/CDV-H protein	Host receptor (SLAM)	CDV/host receptor (SLAM) combi-nation	Predictions for fusion assay (FA)	Predictions for virus titer assay (TA)	Results: evidence for specialist traits	Results: evidence for generalist traits
Domestic dog strain	Domestic dog	A	specialist:Higher value than B, C, D	specialist:Higher value than B,C,D	FA: yesTA: yes: A>B; no:A∼C, A∼D	
Domestic dog strain	Non-dog	B	specialist:Lower value than A	specialist:Lower value than A	FA: yesTA: yes	
Non-dog strain	Non-dog	C	generalist:Similar value to D, higher value than B and lower value than A	generalist:Similar value to D, higher value than B and lower value than A		FA: yesTA: yes: C>B, C∼D; no: C∼A
Non-dog strain	Domestic dog	D	generalist:Similar value to C, higher value than B and lower value than A	generalist:Similar value to C, higher value than B and lower value than A		FA: yesTA: yes: D>B, D∼C; no: D∼A

Predictions designed for CDV-H proteins were tested in an *in vitro* fusion assay (FA) and for CDV strains tested in an *in vitro* virus titration assay (TA). Strong co-evolution of CDV in a homogeneous environment (domestic dog) should produce strains that express specialist traits. Weak co-evolution of CDV in heterogeneous environments (non-dog carnivores) should produce strains expressing generalist traits. The homogeneous experimental environment is Vero cells expressing domestic dog SLAM. The heterogeneous experimental environment is Vero cells expressing African lion SLAM. The last two columns summarize the experimental results from this study.

## Results

### (a) SLAM sequence analysis

**Figure 1:**
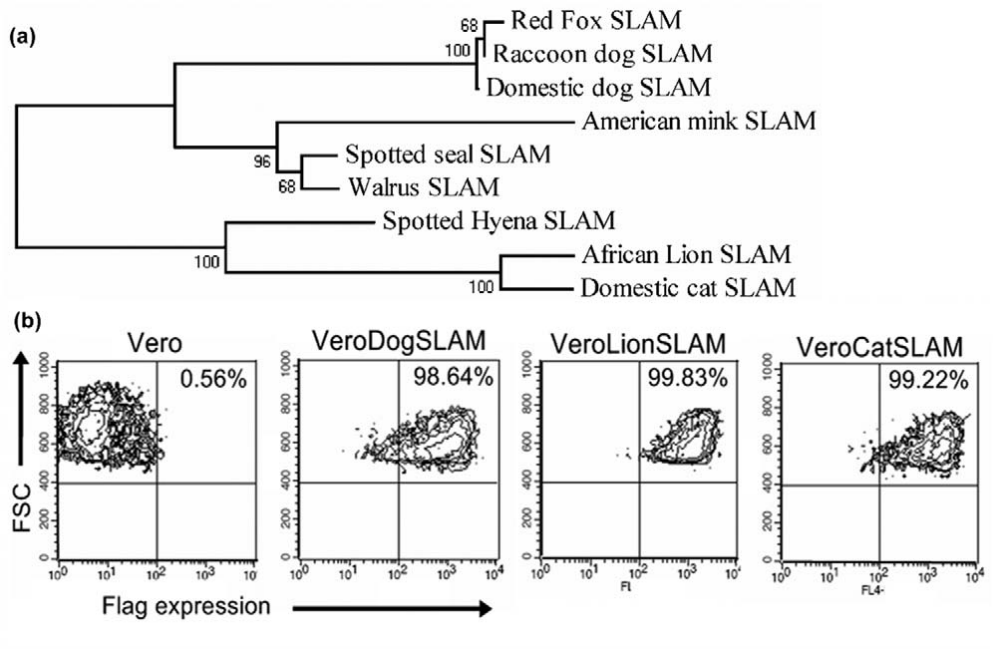
Analysis of SLAM (CD150) receptors from different carnivore species. (a) Phylogenetic analysis of amino acid sequences of carnivore SLAM (CD150) protein (for details see supplementary information-methods), (b) Flow cytometry analysis of parental Vero cells and derived cell lines stably expressing domestic dog, lion, and cat SLAM (CD150) receptors.

A phylogenetic analysis using all currently available complete SLAM amino acid sequence data from carnivores and additionally SLAM sequences from the spotted hyena, African lion and domestic cat produced by this study showed that all sequences from species in the Canidae clustered together, i.e., domestic dog, raccoon dog (*Nyctereutes procyonoides*) and red fox (*Vulpes vulpes*) (figure 1a). These canid SLAM sequences shared a high identity (99.1–99.7%), and far lower identities with SLAM sequences from the spotted seal (*Phoca largha*; 85.2–85.8%) in the Phocidae, the walrus (*Odobenus rosmarus*: 85.2–85.8%) in the Odobenidae, two species of the Felidae, the African lion and domestic cat (73.7–74.9%), one species of the Mustelidae, the American mink (*Neovison vison*, 79.6–79.9%), and one species of the Hyaenidae, the spotted hyena (78.1–78.7%). The sequence identity between the spotted hyena SLAM sequence and SLAM sequences from felids was 85.3%. The lion and domestic cat SLAMs shared a high (96.3%) amino acid sequence identity and formed a cluster phylogenetically distant to canid SLAM sequences (figure 1a). Alignment of the complete SLAM amino acid sequence is presented in the supporting information (figure S1).

### (b) Nuclei per Syncytium

**Figure 2:**
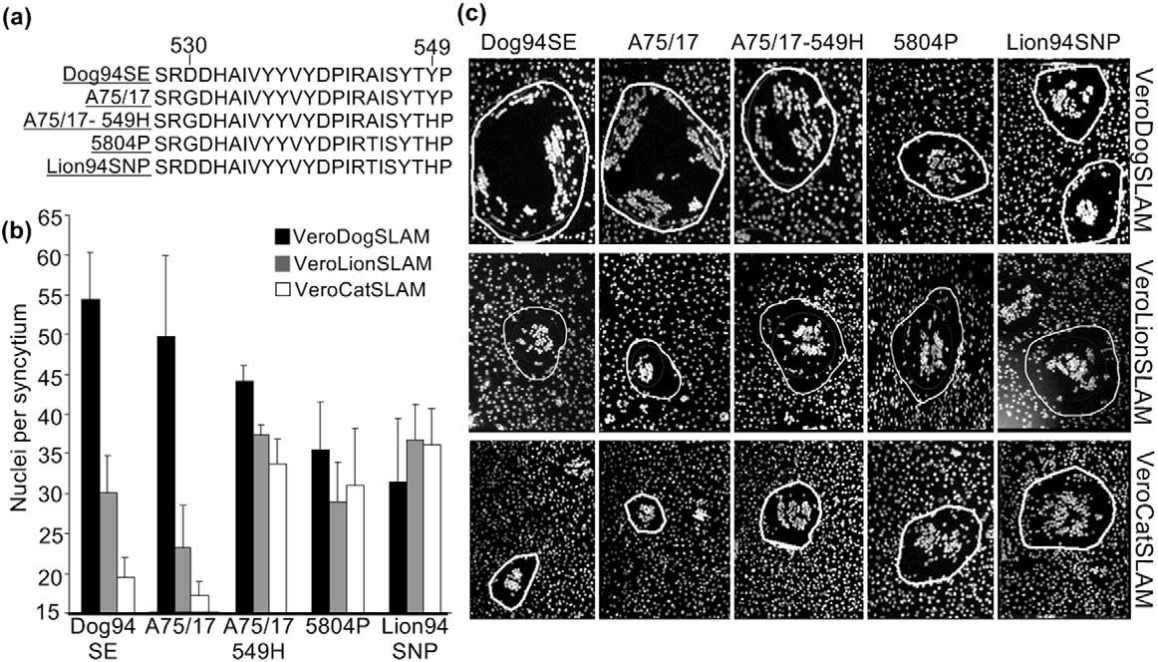
Characterization of the CDV-H protein from CDV strains and their interactions with carnivore SLAM receptors. (a) Alignment of partial SLAM binding region of CDV-H protein sequences of the strains used in this study. Amino acids 528–550 are shown, and the amino acid at position 549 for each strain is indicated. (b) Variation in the extent of syncytia formation (mean number of nuclei per syncytium) induced by proteins from two domestic dog strains (*Dog94SE* and *A75/17*), two non-dog strains (*5804P* and *Lion94SNP*) and one CDV-H protein mutated at position 549 (*A75/17-549H*) in domestic dog (black bars), African lion (grey bars) or domestic cat SLAM-expressing cells (white bars). Error bars indicate standard deviations of six independent experiments. (c) Appearance of syncytia upon expression of CDV-H and CDV-F proteins from two dog strains (*Dog94SE* and *A75/17*), two non-dog strains (*5804P* & *Lion94SNP*) and one mutated CDV-H protein coding 549H (*A75/17-549H*) in domestic dog SLAM, African lion SLAM and domestic cat SLAM cells. Photos were taken 12 hours post transfection at 100× magnification.

The mean number of nuclei per syncytium (MNN per syncytium) significantly varied between CDV-H proteins of different strains (ANOVA-type statistic, ATS = 11.81; degrees of freedom, df = 2.19, ∞; p<0.0001; figure 2b and 2c) and receptor cell lines (ATS = 39.25; df = 2, 15; p<0.0001), and this change in ability across receptor cell lines depended on strain identity (ATS = 11.81; df = 4.38, ∞; p<0.0001). As expected from the lack of clinical symptoms in domestic cats, the MNN per syncytium was significantly smaller in domestic cat SLAM cells (figure 2b, white bars) than in lion SLAM cell lines (figure 2b, grey bars) across CDV-H proteins (ATS = 42.43; df = 1, 10; p<0.0001).

The MNN per syncytium with CDV-H proteins of domestic dog strains *Dog94SE* and *A75/17* significantly changed between receptor cell lines (ATS = 74.97; df = 1.98, 14.7; p<0.0001). Consistent with predicted specialist traits (table 1), post-hoc comparisons demonstrated a significantly higher MNN per syncytium with CDV-H proteins of both domestic dog strains in domestic dog SLAM (figure 2b, black bars) than in lion SLAM cell line (ATS = 67.11; df = 1, 8.90; p<0.0001; figure 2b, grey bars) or domestic cat SLAM cell line (ATS = 60.53; df = 1, 9.54; p<0.0001; figure 2b, white bars). The CDV-H proteins of strains *Dog94SE* and *A75/17* also induced a significantly higher MNN per syncytium in the domestic dog SLAM cell line (figure 2b, black bars) than CDV-H proteins of strains *5804P* and *Lion94SNP* and the mutated CDV-H protein *A75/17-549H* (contrast analysis, ATS = 13.12; df = 1, 5; p = 0.0076, figure 2b, black bars).

Consistent with predicted generalist traits (table 1), CDV-H proteins of strains *5804P*, *Lion94SNP* and mutant *A75/17-549H* showed similar MNN per syncytium in cells expressing SLAM of the two known CDV host species i.e., in lion SLAM and domestic dog SLAM cells (ATS = 1.71; df = 1, 8.93; p = 0.22, figure 2b grey and black bars). The CDV-H proteins of the non-dog strains *5804P* and *Lion94SNP*, and the mutant CDV-H protein *A75/17-549H* induced significantly higher MNN per syncytium in lion SLAM and domestic cat SLAM cell lines than those of the two domestic dog strains *Dog94SE* and *A75/17* (contrast analysis, ATS = 8.63; df = 1, 9.92; p<0.0001, figure 2b grey and white bars).

Replacing residue Y at site 549 by H in the CDV-H protein of domestic dog strain *A75/17* created the experimental CDV-H protein *A75/17-549H*. This single substitution reduced the MNN per syncytium in cell lines expressing domestic dog SLAM receptors and increased the MNN per syncytia in cells expressing the lion SLAM receptors (contrast analysis of *A75/17* against *A75/17-549H*, ATS = 6.20; df = 1, 5.98; p = 0.047; figure 2b). The relative marginal effects on MNN per syncytium by CDV-H proteins from different CDV strains on cells expressing SLAM receptors from different carnivore species are illustrated in figure S5a.

### (c) Effect of SLAM on virus induced syncytia formation

**Figure 3:**
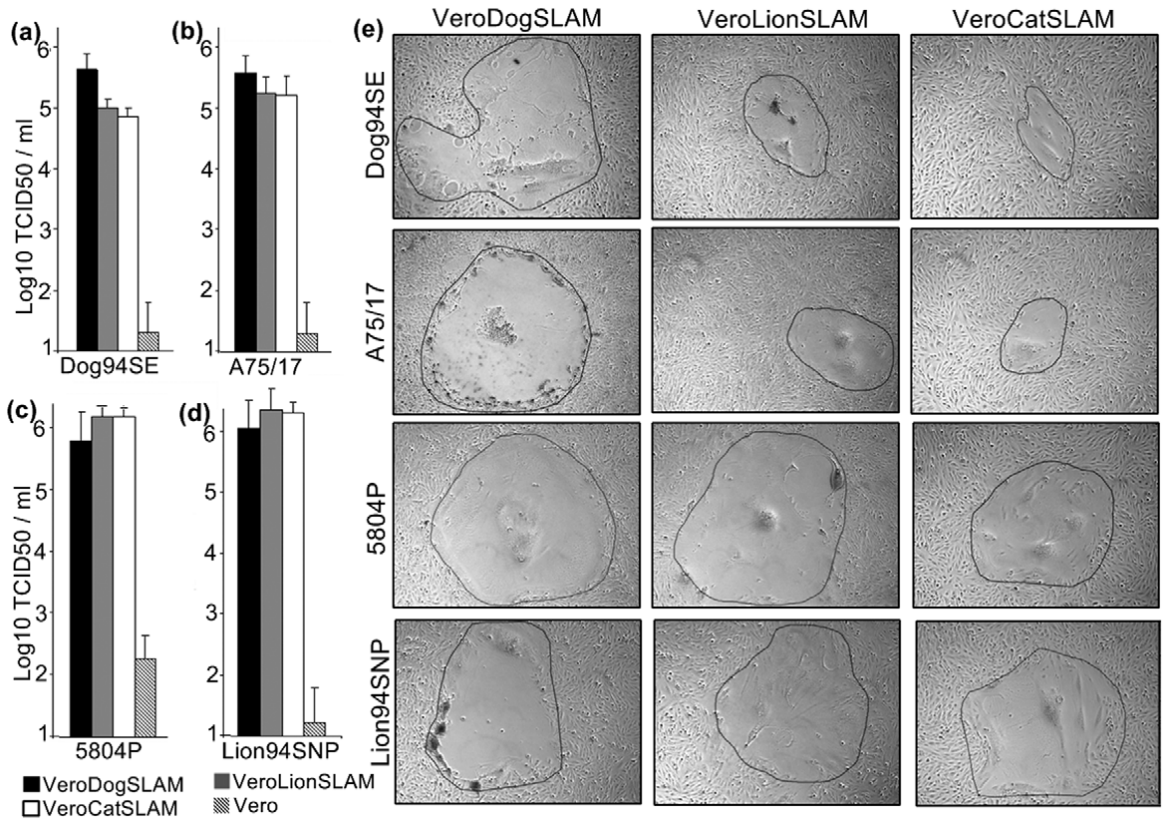
Virus titers and syncytia formation produced by parental Vero cells and Vero cells expressing SLAM receptors of different hosts infected with dog (*Dog94SE* and *A75/17*) and non-dog CDV strains (*5804P* and *Lion94SNP*). Virus titers produced by domestic dog strains (a) *Dog94SE* and (b) *A75/17*, and non-dog CDV strains (c) *5804P* and (d) *Lion94SNP* after 60 h of growth on cells expressing SLAM receptors from different host species. Cells expressing domestic dog SLAM (black bars), African lion SLAM (grey bars), domestic cat SLAM (white bars) and Vero (SLAM negative) cells (hatched bars). Titers were determined using the limited dilution method and expressed as log_10_ TCID_50_/ml. Values shown represent the mean of six independent experiments; error bars indicate standard deviations. (e) The syncytia produced by infection with four CDV strains used in this study on domestic dog, African lion and domestic cat SLAM-expressing cell lines. Photographs were taken 40 h after infection using phase contrast at 100× magnification.

Visual comparison of the size of syncytia formed when domestic dog strains (*A75/17* and *Dog94SE*) and non-dog strains (*P5804* and *Lion94SNP*) were grown on cells expressing SLAM of different species revealed that domestic dog strains induced larger syncytia when grown on cells expressing dog SLAM than in cells expressing lion or cat SLAM. In contrast, the non-dog strains produced similar-sized syncytia in cell lines expressing dog, lion and cat SLAMs (figure 3e). Upon infection of Vero (SLAM negative) cells with any of the CDV strains used in the study, no syncytia were detected during 60 h post infection (data not shown).

### (d) Virus production

Virus titers at 60 h post-infection significantly varied between different strains (ATS = 14.84; df = 2.27, 20.2; p<0.0001); the magnitude of this variation significantly depended on receptor cell lines (ATS = 6.67; df = 3.38, 20.2; p<0.0001; figure 3a–3d).

Post hoc comparisons demonstrated that in line with predicted specialist traits (table 1), the domestic dog strains (*Dog94SE*; *A75/17*) produced higher virus titers in cell lines expressing domestic dog SLAM receptors than in cell lines expressing lion SLAM or cat SLAM (ATS = 6.44; df = 2, 15; p = 0.0096; figure 3a and 3b black bars compared with grey and white bars, respectively). In contrast to predicted specialist traits, dog strains did not produce higher titers than non-dog strains in cell lines expressing domestic dog SLAM receptors (ATS = 0.98, df = 1, 5; p = 0.37; black bars of figure 3a and 3b compared with black bars of figure 3c and figure 3d).

In line with predicted generalist traits, both non-dog strains replicated to similar titer levels in cell lines expressing dog and lion SLAM (ATS = 5.08, df = 1, 7.93; p = 0.055; figure 3c and figure 3d, comparing black and grey bars). In line with both predicted generalist and specialist traits (table 1) both non-dog strains replicated to higher titer levels than domestic dog strains in cell lines expressing lion SLAM and cat SLAM (ATS = 30.87, df = 2.02, 16.8; p<0.0001; grey and white bars of figure 3c and 3d compared with grey and white bars of figure 3a and 3b).

Virus titers of different CDV strains produced by cells expressing SLAM receptors from different carnivore species are illustrated as relative marginal effects in figure S5b.

## Discussion

This study presents evidence consistent with the idea that strong co-evolution of CDV strains in the globally large, homogeneous domestic dog population favored CDV-H proteins specialized to bind to SLAM (CD150) cell receptors of domestic dogs, whereas weak co-evolution in the heterogeneous environment of different carnivore species favored generalist strains less well adapted to domestic dog receptors but able to bind to this cell receptor in a broad range of carnivore species (figure 2). In support of this idea our analysis of SLAM amino acid sequences from carnivores revealed a high similarity (99%) between sequences from three species in the Canidae, and the predicted far lower similarities (72.9–85.2%) between these canid SLAM sequences and SLAM sequences from six species in other carnivore families (Felidae, Hyenidae, Mustelidae and Phocidae figure 1a). Results from the syncytia formation assay provided *in vitro* evidence consistent with the predicted expression of specialist or generalist traits by CDV-H proteins from CDV strains of domestic dogs and non-dog species, respectively (Table 1). Results from the virus titer assay which measured trait expression post cell entry were also mostly consistent with these predictions. Finally, the expected decline in the expression of specialist traits and the increase in the expression of generalist traits by the mutant CDV-H protein (*A75/17-549H*) in which residue H typical of non-dog strains at site 549 replaced residue Y, characteristic of domestic dog strains such as the parent strains *A75/17*, provided evidence of antagonistic pleiotropy, and the involvement of the residue (Y or H) at site 549 [46], [49] as one possible factor influencing CDV host-species range.

Our phylogenetic comparison of amino acid sequences of SLAM (CD150) receptors from species in the Canidae compared to species in other carnivore families (figure 1a) is consistent with the suggestion that CDV strains well adapted to bind to domestic dog SLAM receptors may bind less well to SLAM receptors in hosts from other carnivore families. Currently, the only known receptor for CDV is SLAM (CD150) although CDV is thought to use other receptors, particularly for epithelial cell entry. The theoretical co-evolutionary framework we have applied to SLAM (CD150) could also be tested on alternative CDV cell receptors when these are identified.

Theoretically, specialists should have higher fitness in the homogeneous environment to which they are adapted than generalists. In line with this prediction we found significantly higher performance by both tested CDV-H proteins from domestic dog strains (in terms of MNN per syncytium) in cells expressing domestic dog SLAM than in cells expressing African lion SLAM which is a non-dog host (figure 2b), and domestic dog strains produced higher virus titers in cells expressing domestic dog SLAM than in cells expressing African lion SLAM (figure 3a and 3b). These results provide *in vitro* evidence that the tested CDV-H proteins from domestic dog strains expressed specialist traits. Furthermore, consistent with expectation that a specialist should perform less well in host environments to which it is less well adapted, both CDV-H proteins from domestic dog strains performed less well in cells expressing African lion SLAM than in cells expressing domestic dog SLAM (figure 2b) and produced lower virus titers in cells expressing lion SLAM (figure 3a and 3b).

Consistent with the prediction that generalists should perform less well than a specialist in the environment to which the specialist is adapted, both CDV-H proteins from non-dog strains performed less well (in terms of MNN per syncytium) than both CDV-H proteins from domestic dog strains in cells expressing domestic dog SLAM (figure 2b). However, both non-dog strains produced viral titers in cells expressing domestic dog SLAM similar to those produced by domestic dog strains in cells expressing domestic dog SLAM (figure 3a and 3b). Generalists should perform better than a specialist in environments except the one to which a specialist is particularly well adapted. As expected, CDV-H proteins from non-dog strains performed significantly better (in terms of MNN per syncytium) in cells expressing non-dog SLAM than CDV-H proteins from domestic dog strains in cells expressing non-dog SLAMs (figure 2b) and both non-dog strains produced higher viral titers in cells expressing non-dog SLAM than domestic dog strains produced in cell lines expressing lion SLAM or domestic cat SLAM (figure 3c and 3d). Results of the virus titer assay indicated that factors post cell entry also influenced replication of tested strains.

Although generalists are expected to have a lower fitness than specialists in the environment to which the specialists are adapted, the fitness of generalists should be approximately the same in all environments, including the environment to which a specialist is adapted. Consistent with this prediction, CDV-H proteins of both non-dog strains produced similar MNN per syncytium in cell lines expressing domestic dog SLAM, African lion SLAM and domestic cat SLAM (figure 2b) and non-dog strains produced similar viral titers in cell lines expressing domestic dog SLAM, African lion SLAM and domestic cat SLAM (figure 3c and 3d).

Interestingly, an exceptionally low MNN per syncytium was produced by CDV-H proteins from both domestic dog strains in cells expressing domestic cat SLAM (i.e., SLAM of a species that does not develop clinical CDV), whereas significantly higher values were produced by CDV-H proteins from both non-dog CDV strains in cells expressing domestic cat SLAM. Furthermore, the CDV-H protein from the mutant domestic dog strain *A75/17-549H* produced a significantly higher MNN per syncytium than the CDV-H protein from the parent strain (*A75/17*) (figure 2b) in cells expressing domestic cat SLAM. Also, both non-dog strains produced significantly higher viral titer levels than both dog strains in cells expressing domestic cat SLAM. As domestic cats are not known to exhibit pathological effects of CDV infection, it is likely that immune responses clear CDV before disease is manifested in domestic cats.

Taken together, results from both assays provide considerable evidence for the expression of specialist traits by domestic dog CDV strains (and their CDV-H proteins) and generalist traits in non-dog strains (and their CDV-H proteins). Although our *in vitro* experiments provide strong support for the presence of specialist and generalist traits in CDV strains, they do not demonstrate that these traits are expressed *in vivo*. Even so, we would argue that significant differences in the prevalence of CDV genotypes in dog and non-dog hosts worldwide suggests that this is likely [46], [49].

McCarthy *et al.* [46] proposed that the replacement of residue Y by H at site 549 in the SLAM binding region of the CDV-H protein permitted CDV to spread from domestic dogs to other carnivore species. This hypothesis, within the evolutionary framework of specialists and generalists, suggests that the experimental substitution Y549H in a domestic dog strain would create a mutant expected to express traits more similar to generalist non-dog strains than the specialist traits of dog strains. Our results conformed to this expectation as the mutant CDV-H protein from strain *A75/17-549H* performed significantly less well than the CDV-H protein of the parent domestic dog strain (*A75/17*) in cells expressing SLAM from domestic dogs. Also, as predicted, CDV-H protein of the mutant strain (*A75/17-549H*) performed significantly better than the CDV-H protein from the parent virus in cell lines expressing lion SLAM or domestic cat SLAM. Thus, the residue substitution Y549H caused a significant decline in the expression of specialist traits and an increase in the expression of generalist traits. These results are consistent with the idea that specialist traits that emerge from strong co-evolution in a homogeneous environment entail the cost of reduced fitness in the heterogeneous environments, and that this trade-off is a result of antagonistic pleiotropy [3], [5], [8].

Our results provide evidence that one amino acid site (i.e., site 549) on the CDV-H protein influences the efficiency by which different CDV strains enter cells of domestic dog and other carnivore hosts. Even so, it is probable that additional sites may also influence the ability of CDV to enter the cells of different host species. Specific residues at site 530 in the CDV-H protein were suggested to be an adaptation of CDV to non-domestic dog hosts [46] but recent genetic evidence suggests this site is conserved within CDV lineages [49].

Modeling the transmission of infectious viruses that utilize multiple host species is extremely complex [54], [55] and most models of CDV transmission consider just one host species [56], [57]. There are many disease models that consider how infection by one strain of a pathogen alters host reaction to another [58]–[63]. These models mostly consider cross- immunity between strains and antibody-dependent enhancement of infection. Our results suggest that one key level of complexity hitherto ignored in models of CDV in multi-host carnivore communities is the incorporation of the effect of strains that differ in their fitness in specific host species. Traditionally, CDV infections in wildlife were assumed to result from spill-over infection from domestic dogs. The results of our phylogenetic analysis suggest that spill-over of domestic dog strains to other species within the family Canidae is more likely than to non-canid species.

Most discussions of CDV evolution assume that this multi-host virus appeared relatively recently in domestic dogs [37] and then spread to non-dog host species [64]. Although our results do not contradict this interpretation, a plausible alternative evolutionary route could be that CDV originated and was maintained as a generalist virus in a wide range of carnivore species. Specialist CDV strains would be expected to emerge when the size and density of a domestic dog population was sufficient to permit strong host species-specific selection for the emergence and maintenance of specialist strains. Generalist strains would be expected to persist and out-perform specialist strains in multi-host environments and specialist strains to out-perform generalist ones in the homogeneous domestic dog host environment.

Taken together we present evidence for the expression of specialist and generalist traits in CDV strains. Our results have important implications for the understanding of the evolution and epidemiology of this multi-host infectious virus in carnivores, and to the risk of infection posed by specialist and generalist strains to domestic dog and wild carnivore populations.

## Materials and Methods

### (a) Cell lines and viruses

Vero (African green monkey kidney) cell lines ATCC CCL-81 (American Type Culture Collection, USA) and all stable cell lines produced in this study were maintained in Earle's Minimal Essential Medium (MEM) (Invitrogen, USA) supplemented with 5% of fetal calf serum (FCS) (Invitrogen, USA).

The four CDV strains used in this study included two domestic dog strains (*Dog94SE* and *A75/17*), one strain from an African lion (*Lion94SNP*) and one ferret (*Mustela putorius furo*) strain *5804P* [65]. Strain *Dog94SE* was isolated from a domestic dog close to the Serengeti ecosystem, Tanzania, in 1994, the *Lion94SNP* isolate was obtained from a lion during a CDV outbreak in wildlife in the Serengeti National Park, Tanzania, in 1994. As these two strains were obtained in the same year and within the same geographical area of Tanzania, from a domestic dog and non-dog host, they are well suited to test for specialist and generalist traits, as the null hypothesis would predict CDV strains from the same geographical area and time-frame should perform equally in our assays. These two CDV isolates from Tanzania were initially isolated in dog PBMCs in 1994 and then were kept at −80°C until this study. Strain *A75/17* was recovered from a domestic dog [64] and maintained by passaging it on VeroDogSLAM cells. Strain *5804P* is a domestic ferret (*Mustela putorius*) adapted CDV strain [65] derived when the Vero cell-adapted wild-type strain *5804* was passaged three times through domestic ferrets. The wild-type strain *5804* was initially isolated from a CDV infected domestic dog in dog PBMCs. Adaptation to Vero cells probably explains why strain *5804P* performed marginally better (figure 3c) than other strains tested (figure 3a,b,d) in SLAM-negative Vero cells in the virus titer assay.

These two additional strains provided a domestic dog and a non-dog adapted strain from different CDV genetic lineages. Prior to the experiments, strains *Dog94SE*, *A75/17* and *5804P* were passaged once on Vero cells expressing the domestic dog SLAM receptor, and strain *Lion94SNP* was amplified on Vero cells expressing the lion SLAM receptor. Virus titers were determined by the standard limiting dilution method, and 50% tissue culture infectious doses (TCID_50_) were calculated [66].

### (b) Construction of protein expression vector expressing CDV-H and CDV-F proteins

The CDV-H and CDV-F protein genes from strain *Lion94SNP* (JN812975 and JN812977), strain *Dog94SE* (JN812976) and strain *A75/17* (AF112189 and AF112188) were cloned directly into the pCG eukaryotic expression vector using BamHI and SphI restriction enzymes, yielding pCG-*lion94SNP*-CDV-H, pCG-*lion94SNP*-F, pCG-*Dog94SE*-CDV-H, pCG-*A75/17*-CDV-H and pCG-*A75/17*-CDV-F. The pCG protein expression vectors containing the *5804P* CDV-F and CDV-H genes (AY386316) were published previously [38]. Protein expression vector pCG-*A75/17*-CDV-H was used for site-directed mutagenesis at amino acid position 549 using the QuickChange Site-Directed Mutagenesis Kit (Agilent Technologies, USA) applied according to manufacturer's instructions and employing primers *A75/17*-Y549H-S (5′-ccgggcgatttcttatacgcacccatttagactaacta-3′) and *A75/17*-Y549H-AS (5′-tagttagtctaaatgggtgcgtataagaaatcgcccgg-3′). This resulted in an additional protein expression vector coding histidine (H) at position 549 (pCG-*A75/17*-549H-CDV-H) which was labeled as *A75/17-549H* in the results for the syncytia formation assay (figure 2b and 2c).

### (c) Construction of protein expression vectors expressing SLAM receptors and generation of SLAM expressing cell lines

Peripheral blood mononuclear cells (PBMC) were extracted from blood samples of spotted hyenas and African lions collected during routine veterinary examination of zoo animals in Germany, and domestic cat blood was obtained from healthy domestic cats sampled in a veterinary clinic. SLAM genes were amplified from concanavalin A (Con A) stimulated PBMCs and sequenced. SLAM open reading frames without the signal peptide sequence were cloned into a pCG vector already carrying an Igκ signal peptide, a FLAG tag at the amino-terminus of the inserted protein and the zeocin resistance gene [67] (see figure S2). The nucleotide sequences of all protein expression vectors were confirmed by sequencing. Vero cell lines were transfected with the respective SLAM protein expression vector using the Fugene transfection reagent (Roche, Germany). SLAM-expressing cells were selected using 1 mg/ml of Zeocin (Invitrogen, USA), resistant cells were subcloned and clones with similar SLAM surface-expression levels selected for further experiments (figure 1b). For details see supporting information - methods.

### (d) Syncytia formation assay

Vero cells expressing domestic dog, African lion, or domestic cat SLAM were seeded in 6-well plates and each well was co-transfected using the 3 µl of Fugene transfection reagent (Roche, Germany) with five different CDV-H and CDV-F protein expression vector combinations: (1) pCG-*A75/17*-CDV-H+pCG-A75/17-CDV-F (*A75/17*), (2) pCG-*Dog94SE*-CDV_H+pCG-*Lion94SNP*-CDV-F (*Dog94SE*), (3) pCG-*A75/17*-549H-CDV-H+pCG-*A75/17*-CDV-F (the experimentally mutated - *A75/17-549H*), (4) pCG-*5804P*-CDV-H+pCG-*5804P*-F (*5804P*), (5) pCG-*Lion94SNP*-CDV-H+pCG-Lion94SNP-CDV-F (*Lion94SNP*), using 0.15 µg DNA from each protein expression vector per well. Twelve hours after transfection, cells were fixed, permeabilized, and nuclei were visualized with Hoechst 33342 trihydrochloride trihydrate (Invitrogen, USA). Pictures were taken using a Zeiss AxioVert S100 microscope (Carl Zeiss AG, Germany) and recorded with an Axiocam CCD camera (Carl Zeiss AG, Germany). For each cell line, six independent transfection experiments were performed. For each experiment, five random images were taken of each well, syncytia were marked and nuclei were counted in a blinded fashion, counting the nuclei within a total of 65–75 syncytia for each cell type and protein expression vector combination (i.e. a total of 390–450 syncytia per combination of cell type and protein expression vector).

As a control for the syncytia formation assay, all three cell lines were transfected with empty pCG plasmids and individually with pCG plasmids encoding CDV-H or CDV-F genes. None of the control experiments produced syncytia within 24 h (data not shown). The presence of SLAM on target cells was shown as important for CDV H and F induced syncytia formation (figure S3). Furthermore, to ensure that the CDV-F protein did not influence the extent of syncytia formation measured by the assay, different CDV-F proteins were combined with CDV-H proteins used in this study. No differences were observed in the MNN per syncytia when we expressed different CDV-F proteins with the same CDV-H protein (figure S4). This is consistent with previous reports according to which the size of syncytia formation mainly depended on the CDV-H protein and its ability to bind its cognate receptor, and is not significantly affected when CDV-F proteins from different strains or even different morbilliviruses are applied [38], [45], [68].

### (e) Virus growth

Vero cells expressing domestic dog, African lion, or domestic cat SLAM were seeded in 6-well plates at 10^6^ cells/well and infected 12 h later with virus strains *A75/17*, *Dog94SE*, *5804P* or *Lion94SNP* at a multiplicity of infection (MOI) of 0.01. To evaluate the fusion efficiencies of domestic dog and non-dog CDV strains, the syncytia were photographed 40 h post infection (figure 3e) and the cells were harvested 20 h later at 60 h post infection for virus titration. Viral titers were determined using the limiting dilution method and expressed as TCID_50_. As a control, Vero (SLAM negative) cells were infected following the same procedure with virus strains *A75/17*, *Dog94SE*, *5804P* or *Lion94SNP* and viral titers determined following the same method. Viral titers in all cell lines were extremely low (figure 3a–3d).

### (f) Statistical analysis

The experiments performed for both assays corresponded to a split-plot design of independent experiments. We used the ‘nonparametric marginal model’ (NMM) [69], [70] to assess the statistical significance of the results of the fusion assay (six replicates of mean number of nuclei per syncytium per experiment) and the viral titer assay (six replicates of viral titers per experiment) produced by Vero cell lines with different SLAM receptors and infected with different CDV-H proteins and different strains respectively. The NMM has the advantage of not making any assumptions about data distribution, only uses the empirical distribution of the data and can be used to analyze factorial experiments of arbitrary complexity (corresponding to general linear models or GLMs). The NMM is superior to well-known parametric models such as GLMs which make many assumptions about data distribution and require properties rarely fulfilled by data sets in medicine or biology with small sample sizes.

Testing for significant differences between the effects of different factors such as type of receptor or type of strain was performed by employing an analysis of variance (ANOVA)-type statistic (ATS) [69], [70]. The ATS approximately follows an F-distribution (for details see [69], p.32). We also used the NMM to calculate relative marginal effects to provide a graphical representation of the effect size when changing between factor categories. A relative marginal effect of 0.5 for a given factor category indicates that it is equivalent to the mean. If the effect is lower than 0.5, then the values for that category tend to take on smaller values than the results for all factor categories. The smaller the relative marginal effect, the stronger is the tendency to smaller values with respect to the average distribution. If the effect is larger than 0.5, then the values for that factor category tend to take on larger values than the results for all factor categories. For further details see references [69] and [70] and the supplementary methods.

## Supporting Information

Methods S1 (DOC)

Figure S1 (DOC)

Figure S2 (DOC)

Figure S3 (DOC)

Figure S4 (DOC)

Figure S5 (DOC)
