# Supplementary material for: Antagonistic Pleiotropy and Fitness Trade-Offs Reveal Specialist and Generalist Traits in Strains of Canine Distemper Virus
Source: PLoS One. 2012 Dec 11;7(12):e50955. doi: 10.1371/journal.pone.0050955 (PMC3519774; doi:10.1371/journal.pone.0050955)
Supplement: Methods S1 — (DOC) [file pone.0050955.s001.doc]

**SUPPorting MethodS**

**1. Sequencing and phylogenetic analysis of SLAM**

To amplify the SLAM open reading frames of the respective species, fresh PBMCs from domestic dog, spotted hyena, African lion and domestic cat were separated by gradient centrifugation and stimulated with concanavalin A (Sigma, USA) for 48 h. RNA from stimulated PBMCs were isolated using RNeasy mini kit (Qiagen, Germany). The RNA was reverse transcribed using Superscript III reverse transcriptase (Invitrogen, USA), and SLAM sequences without the signal peptide sequence were amplified using primers 1-8 (table S1) and proofreading polymerase (LongAmp, NEB, USA). The amplicons were cloned into Topo pcr2.1 vector (Invitrogen, USA) and were sequenced using standard M13 primers. The maximum-likelihood method was performed using the Jones-Taylor-Thornton method and gamma (*G*) distributed rates among sites to generate the phylogenetic tree (figure 1a). Available SLAM gene sequences from domestic dog (NM_001003084), red fox (EU678638), raccoon dog (EU678639), spotted seal (AB428368), walrus (AB428369) and American mink (FJ626692) were used for comparison with the SLAM sequences from spotted hyena (JN812974), African lion (JN812972) and domestic cat (JN812973) obtained in this study.

| **Primer**  **No.** | **Primer name** | **Primer sequence 5` - 3`** |
| --- | --- | --- |
| 1. | LionSLAM-fwd | TTTTCCGCGGGAGAGCTTGATGAATTG |
| 2. | LionSLAM-rev | TTTTGCATGCTCAGCTCTCCGGCAGTG |
| 3. | CatSLAM-fwd | TTTTCCGCGGGGAAAGCTTGATGACTTTTCAAGAGGTCAC |
| 4. | CatSLAM-rev | TTTTGCATGCTCAGCTCTCCGGCAGTG |
| 5. | DogSLAM-fwd | TTTTCCGCGGGAGAGCTTGATGAATTGCCCAG |
| 6. | DogSLAM-rev | TTTTGCATGCTCAGCTCTCTGGGAACGTCAC |
| 7 | HyenaSLAM-fwd | TTTTCCGCGGGAGAGCTTGATGAATTG |
| 8 | HyenaSLAM-rev | TTTTGCATGCTCAGCTCTCCGGCAGTG |

**Table S1**. Primers used for amplification of SLAM open reading frames from different carnivore species. Sites for restriction enzymes introduced into SLAM amplicons and used for cloning into expression vectors (pCG) are underlined.

**2**. **Generation of SLAM expressing cell lines**

Domestic cat, African lion, and domestic dog SLAM genes were introduced into an eukaryotic expression vector and stable Vero cell lines were generated. The cell lines were subcloned, and clones with similar expression levels were selected for further experiments (figure 1b). For this purpose, Vero cells were seeded in 6-well plates at 70-80% confluence and transfected with 1µg of the respective SLAM expression plasmid using 4 µl of Fugene HD transfection reagent (Roche, UK). SLAM-expressing cells were selected by constant presence of 1mg/ml of Zeocin selection reagent (Invitrogen, USA) within 14 days, and Zeocin resistant cells were cloned by the limited dilution method. SLAM expression levels of the different clones were determined by flow cytometry analysis (BD FACSCalibur) using the monoclonal mouse anti-FLAG M2 antibody (Sigma, USA) in combination with Alexa Fluor 647-labeled goat anti-mouse IgG antibodies (Invitrogen, USA). Clones with similar expression profiles and at least 94% FLAG-positive cells were chosen for future experiments.

To further quantify expression levels, mean intensities of the fluorescence of FL-4 (FLAG) cells were calculated using FlowJo software (Tree star, Ashland, Oregon, USA). The values were as follows: 10.5 for Vero negative cells, 1062 for VeroDog SLAM, 1744 for VeroCat SLAM and 1584 for VeroLion SLAM respectively. Compared to negative untransfected cells, all three FLAG-transduced cell lines showed high levels of FLAG labeled SLAM as should have been the case.

**3. Quantification of the number of nuclei per syncytium**

We quantified the number of nuclei per syncytium 12h post-transfection because our aim was to determine the number of nuclei in each syncytium, i.e., before two or more syncytia fused. We chose this point in time because our experience showed that between 12h and 24h post-transfection many syncytia that were single and present at 12h post-transfection fused.

**4. Relative marginal effects in the nonparametric marginal model**

Relative effects are termed relative marginal effects if - as in the case here – the entire distribution (and not just the means) of the individual data points for each factor are used to check for changes in the distribution of data points between factor categories, a more general approach than focusing on the mean or other central tendencies. In other words, the graphical illustration (see figure S5) of relative marginal effects is equivalent to a standardization or re-scaling of original measurement values; relative marginal effects therefore permit the direct comparison of effect sizes between different factors and even different dependent variables such as the mean number of nuclei per syncytium or viral titers.
