# Supplementary material for: Antagonistic Pleiotropy and Fitness Trade-Offs Reveal Specialist and Generalist Traits in Strains of Canine Distemper Virus
Source: PLoS One. 2012 Dec 11;7(12):e50955. doi: 10.1371/journal.pone.0050955 (PMC3519774; doi:10.1371/journal.pone.0050955)
Supplement: Figure S1 — (DOC) [file pone.0050955.s002.doc]

**FIGURE S1**


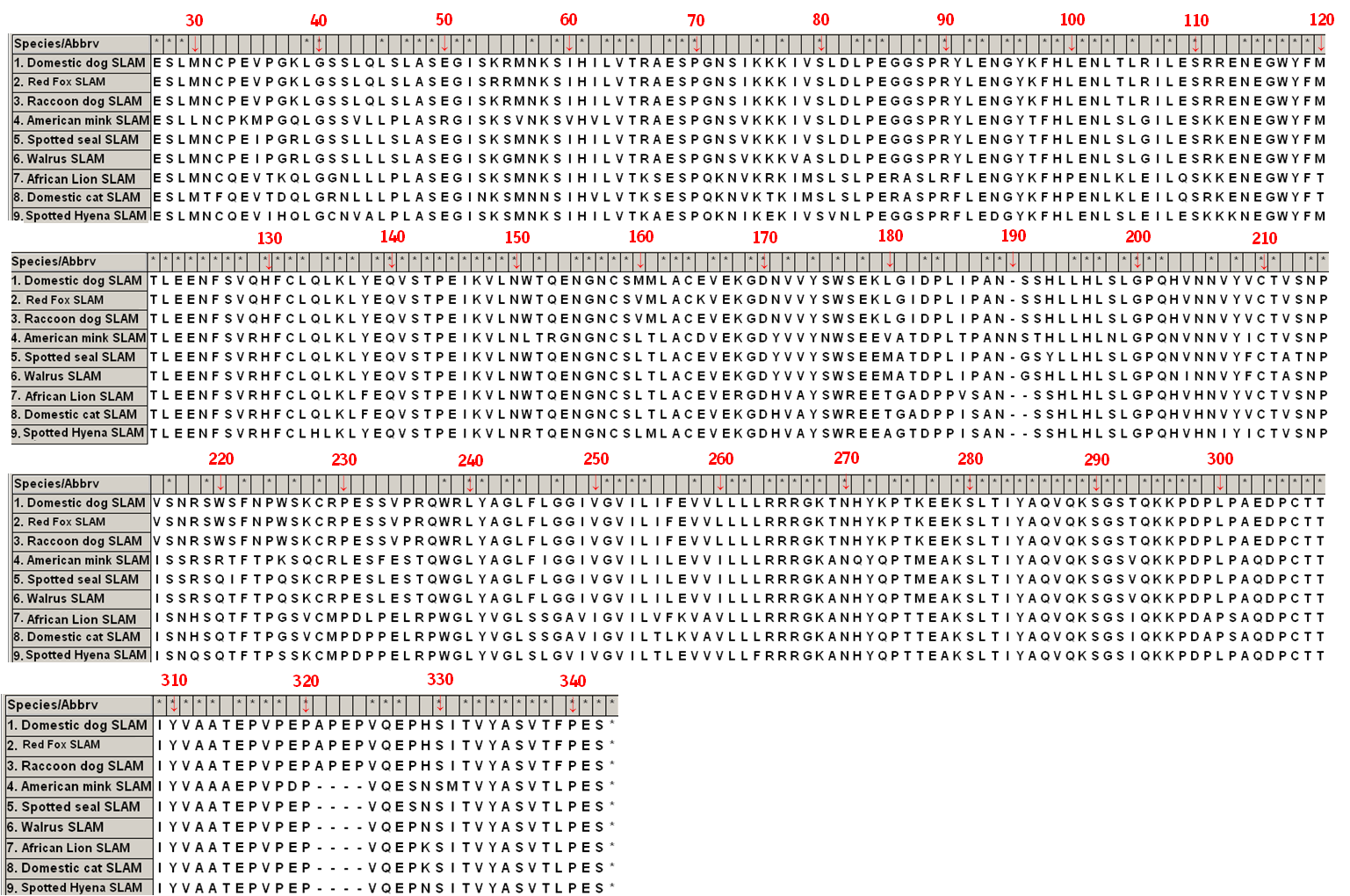


**Figure S1.** Alignment of amino acid sequences of SLAM (CD150) from different carnivore hosts. The alignment of the entire SLAM amino acid sequence (without signal peptide) of nine carnivore species is shown, including deduced SLAM sequences of domestic cat, African lion (Lion) and spotted hyena (Hyena).
