# Supplementary material for: Antagonistic Pleiotropy and Fitness Trade-Offs Reveal Specialist and Generalist Traits in Strains of Canine Distemper Virus
Source: PLoS One. 2012 Dec 11;7(12):e50955. doi: 10.1371/journal.pone.0050955 (PMC3519774; doi:10.1371/journal.pone.0050955)
Supplement: Figure S2 — (DOC) [file pone.0050955.s003.doc]

**FIGURE S2**

**Figure S2.** Construction of pCG mammalian expression vector used for the creation of Vero cells expressing SLAM receptors of different carnivore species. Upstream of the SLAM sequence (lacking the sequence coding the signal peptide) the IgK signal sequence was inserted, followed by the sequence coding FLAG tag associated with the N-terminus of SLAM. Downstream of the SLAM sequence, the zeocin resistance gene (*zeo*) was encoded.
