# Supplementary material for: Antagonistic Pleiotropy and Fitness Trade-Offs Reveal Specialist and Generalist Traits in Strains of Canine Distemper Virus
Source: PLoS One. 2012 Dec 11;7(12):e50955. doi: 10.1371/journal.pone.0050955 (PMC3519774; doi:10.1371/journal.pone.0050955)
Supplement: Figure S3 — (DOC) [file pone.0050955.s004.doc]

**FIGURE S3**


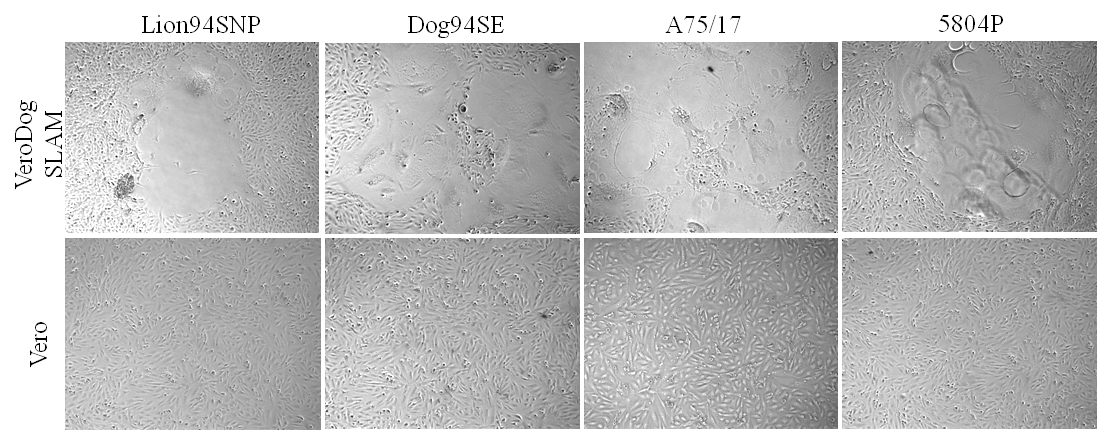


**Figure S3.** The importance of SLAM for syncytia formation. Vero cells which do not express SLAM (lower panels) did not support formation of syncytia upon expression of CDV-H and CDV-F proteins from the strains used in this study. In contrast, Vero cells expressing domestic dog SLAM (upper panels) formed syncytia after transfection with H and F proteins from the tested CDV strains. Photos were taken 14 hours post transfection of plasmids expressing H and F genes of different CDV strains at a magnification of 100x.
