# Supplementary material for: Antagonistic Pleiotropy and Fitness Trade-Offs Reveal Specialist and Generalist Traits in Strains of Canine Distemper Virus
Source: PLoS One. 2012 Dec 11;7(12):e50955. doi: 10.1371/journal.pone.0050955 (PMC3519774; doi:10.1371/journal.pone.0050955)
Supplement: Figure S4 — (DOC) [file pone.0050955.s005.doc]

**FIGURE S4**


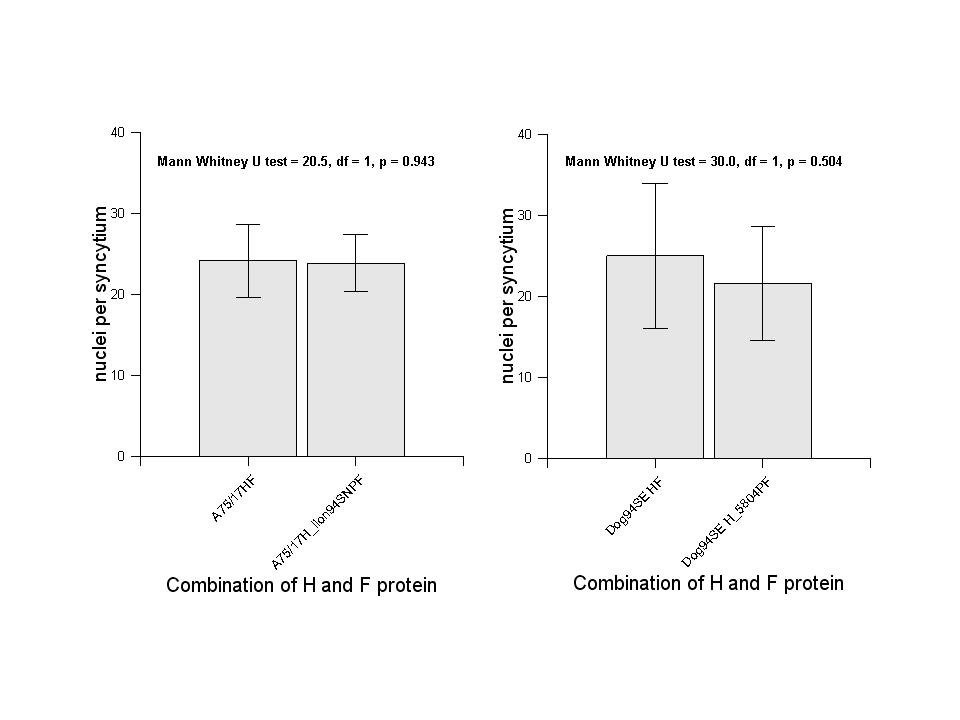


**Figure S4.** Comparison of the number of nuclei per syncytium induced by different combinations of CDV-H and CDV-F protein expressed on VeroCatSLAM cells. Nuclei per syncytium were determined by the same method described in the method section of this article. Values shown represent the mean of at least six independent photos for each plasmid combination, error bars indicate standard deviations.
