# Supplementary material for: Antagonistic Pleiotropy and Fitness Trade-Offs Reveal Specialist and Generalist Traits in Strains of Canine Distemper Virus
Source: PLoS One. 2012 Dec 11;7(12):e50955. doi: 10.1371/journal.pone.0050955 (PMC3519774; doi:10.1371/journal.pone.0050955)
Supplement: Figure S5 — (DOC) [file pone.0050955.s006.doc]

**FIGURE S5**

(a) (b)


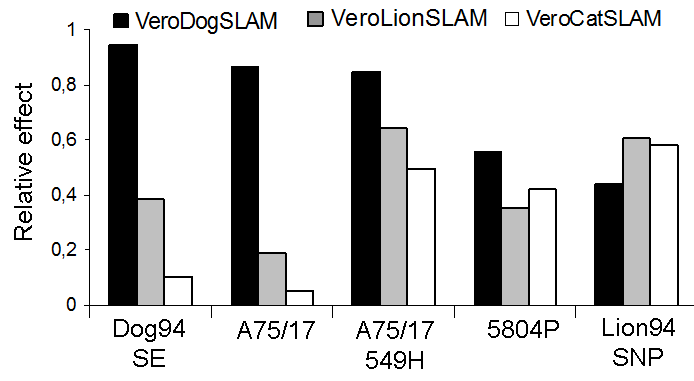

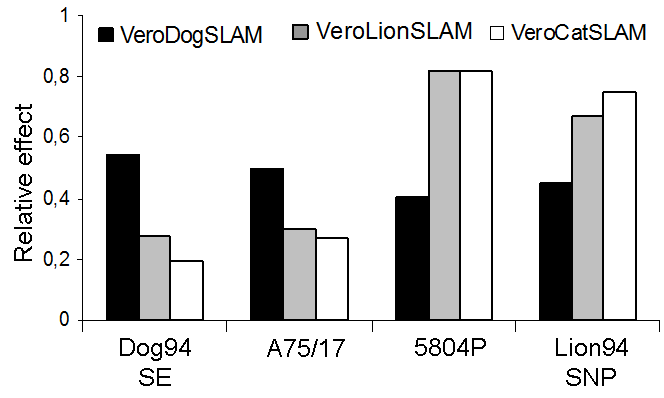


**Figure S5. Relative marginal effects.** (a) Relative marginal effects of the extent of syncytia formation (nuclei per syncytium) by the CDV-H proteins of two domestic dog CDV strains (*Dog94SE* and *A75/17*), two non-canid strains (*5804P* and *Lion94SNP*) and experimentally derived plasmid (*A75/17-549H*) in the three cell lines expressing domestic dog, lion or domestic cat SLAMs. (b) Relative marginal effects of virus titres of two domestic dog strains (*Dog94SE* and *A75/17*) and two non-canid strains (*5804P* and *Lion94SNP*) produced by infection of cell lines expressing domestic dog, lion or domestic cat SLAMs.
